# Identification of a host collagen inducing factor from the excretory secretory proteins of *Trichinella spiralis*

**DOI:** 10.1371/journal.pntd.0006516

**Published:** 2018-11-01

**Authors:** Mi Kyung Park, Hae-Jin Kim, Min Kyoung Cho, Shin Ae Kang, So Young Park, Se Bok Jang, Hak Sun Yu

**Affiliations:** 1 Department of Parasitology School of Medicine, Pusan National University, Yangsan, Republic of Korea; 2 Department of Molecular Biology, College of Natural Sciences, Pusan National University, Busan, Republic of Korea; Istituto Superiore di Sanità, ITALY

## Abstract

**Background:**

In a previous study, we found that *Trichinella spiralis* muscle larva excretory and secretory proteins (ES-P) most likely activate collagen synthesis via TGF-β/Smad signaling, and this event could influence collagen capsule formation.

**Methodology/Principal findings:**

In order to identify the specific collagen inducing factor, ES-P was fractionated by a Superdex 200 10/300 GL column. We obtained three large fractions, F1, F2, and F3, but only F3 had collagen gene inducing ability. After immunoscreening, 10 collagen inducing factor candidates were identified. Among them, TS 15–1 and TS 15–2 were identical to the putative trypsin of *T*. *spiralis*. The deduced TS 15–1 (M.W. = 72 kDa) had two conserved catalytic motifs, an N-terminal Tryp_SPc domain (TS 15-1n) and a C-terminal Tryp_SPc domain (TS 15-1c). To determine their collagen inducing ability, recombinant proteins (rTS 15-1n and rTS 15-1c) were produced using the pET-28a expression system. TS 15–1 is highly expressed during the muscle larval stage and has strong antigenicity. We determined that rTS 15-1c could elevate collagen I via activation of the TGF-β1 signaling pathway *in vitro* and *in vivo*.

**Conclusion/Significance:**

In conclusion, we identified a host collagen inducing factor from *T*. *spiralis* ES-P using immunoscreening and demonstrated its molecular characteristics and functions.

## Author summary

*Trichinella spiralis* can make collagen capsules in host muscle cells during its life cycle, which encapsulates muscle stage larvae. Many investigators have tried to reveal the complex mechanism behind this collagen capsule architecture, and it has been suggested that several serine proteases in excretory-secretory proteins of the parasite are potential collagen capsule inducing factors. In addition, collagen synthesis is activated through the TGF-β/Smad signaling pathway and these events are closely related with protease activated receptor 2 which was activated by various serine proteases. In this study, we isolated and characterized a collagen gene expression inducer from *T*. *spiralis* ES-P using immunoscreening and investigated the candidate protein for its usefulness as a wound healing therapeutic agent.

## Introduction

*Trichinella spiralis* can make collagen capsules in host muscles during their life cycle that surround muscle stage larvae and might protect the larvae from the host immune system. This phenomenon can be understood as the parasite creating a simple structure to protect itself, but when examined closely, numerous different mechanisms are involved in this stage of the parasite’s life. Division of the host muscle cell nucleus, regulation of host cell cycling, huge elevation of host collagen gene expression, and generation of new blood vessels around the collagen capsule are observed during nurse cell formation by *T*. *spiralis* [1–4]. The process of nurse cell formation induces de-differentiation, cell cycle re-entry, arrest of infected muscle cells, and activation, proliferation, and differentiation of satellite cells. These events are very similar to those occurring during muscle cell regeneration and repair [2].

In a previous study, we found that *T*. *spiralis* excretory and secretory proteins (ES-P) most likely activate collagen synthesis via TGF-β/Smad signaling, and this event could influence collagen capsule formation [5]. These events were closely related with protease activated receptor 2 (PAR2), which was activated by various serine proteases [5]. However, the question of which protease in *T*. *spiralis* ES-P has a role in collagen gene expression of host muscle cells is still unanswered. The identification of a specific collagen gene inducer from *T*. *spiralis* could be exploited as a therapeutic and/or cosmetic agent. In this study, we isolated and characterized the collagen gene expression inducer from *T*. *spiralis* ES-P by immunoscreening and investigated the candidate for its usefulness as a wound healing therapeutic agent.

## Materials and methods

### Isolation of muscle larvae and extraction of whole parasite proteins

The *T*. *spiralis* strain (isolate code ISS623) used in this study has been maintained in our laboratory via serial passage in rats. For acquisition of muscle larva, eviscerated mouse carcasses were cut into pieces, followed by digestion in 1% pepsin 1% hydrochloride digestion fluid (artificial gastric juice) for 1 hr at 37°C with stirring. Larvae were collected manually from muscle digested solution under microscopy and washed 6 times with sterile PBS containing 100 μg/ml ampicillin, 5 μg/ml kanamycin and 50 μg/ml tetracyclin. After collection, in order to prevent contamination with the host material, worms were thoroughly and carefully washed several 3 times with PBS. Whole parasite proteins (total extract; TE) was obtained from muscle larva according to previous study [6]. In brief, muscle larva were rinsed in PBS and homogenized in 50 mM Tris–HCl (pH 7.5) with a glass homogenizer. The homogenates were briefly sonicated and then centrifuged for 30 min at 12,000 × g and 4°C. The supernatant (TE) was stored at -20°C.

### Isolation of adult worm and new born larvae (NBL)

Small intestines were removed on the day 6 after infection from infected rat, opened, sliced by 2 cm, washed with PBS, and incubated for 1 hr at 37°C in PBS containing antibiotics. Adult worms were collected on a PBS, washed 3 times with PBS containing antibiotics, and incubated for 24 hrs in serum-free RPMI 1640 medium containing antibiotics. After incubation, NBL were passed through 40 μl nylon mesh (BC falcon, USA) to be separated from adult worms.

### Extraction of ES-P from muscle larvae and fractionation of ES-P

Muscle larvae were isolated from *T*. *spiralis* infected mice (4 weeks after infection) and ES-P from cultured muscle larvae was obtained according to the previously reported method [5]. The ES-P was fractionated using gel filtration chromatography. ES-P (5 mg) in 10 ml PBS was applied to a Superdex 200 10/300 GL column (GE Healthcare, Uppsala, Sweden). The flow rate was 0.25 ml/min. Each 0.5 ml fraction was collected and protein quantity was measured by UV detection at 260 nm. Three big fractions, F1, F2, and F3, were acquired and used for collagen gene inducing experiments (Fig 3A).

### Mouse experiments

**Fig 1:**
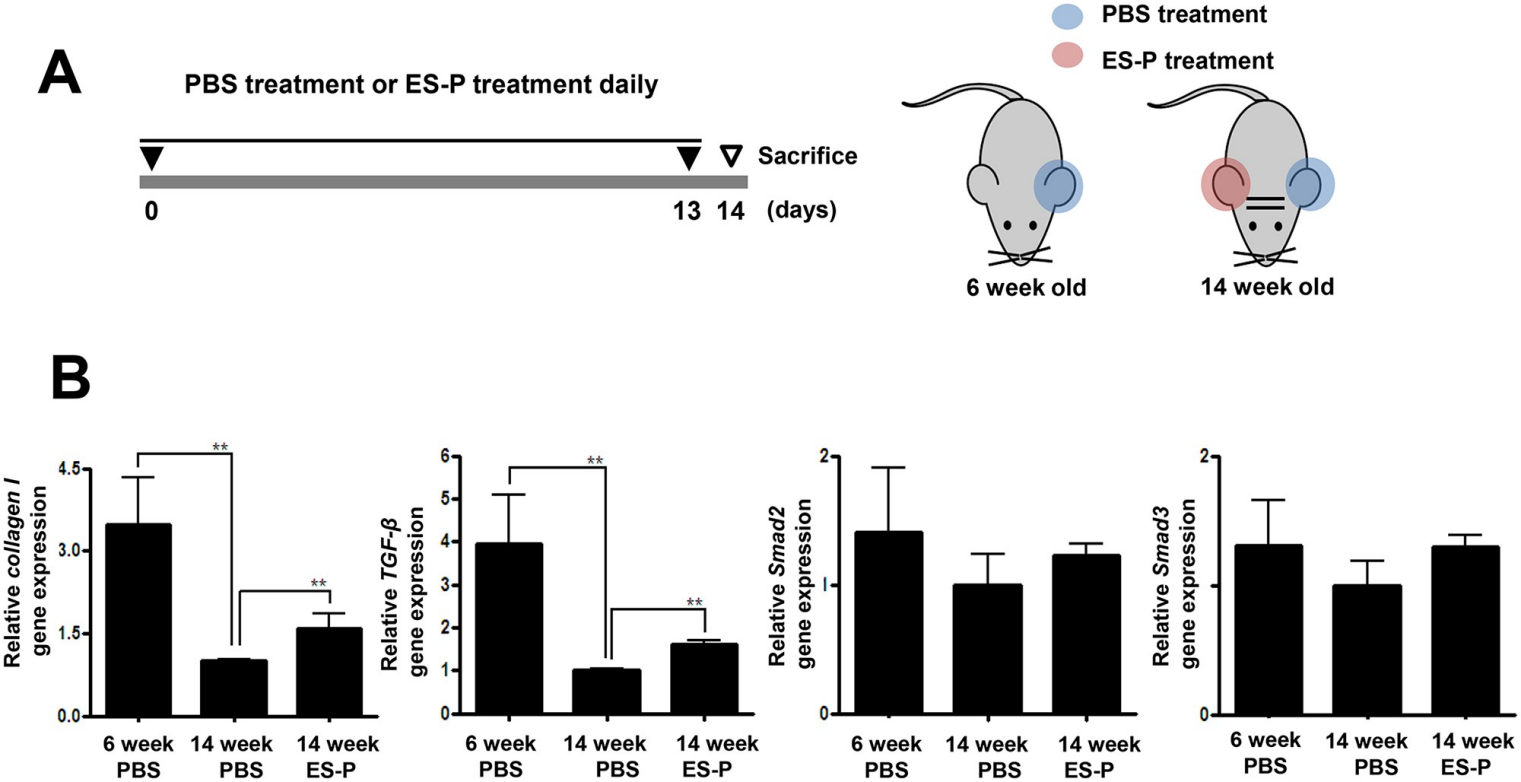
Expression levels of type I collagen, Smad2/3, and TGF-β1 genes after ES-P treatment in aged mice. (A) The left ears of fourteen-week old mice were treated daily with 30 μg *T*. *spiralis* ES-P for 14 days (red color). The right ears of were treated daily with PBS for 14 days as control (blue color). After 14 days, ears were collected. Total RNA was extracted from each ear tissue and cDNA was constructed. (B) The gene expression levels of *collagen I*, *TGF-β1*, and *Smad2/3* were analyzed by real-time PCR. The gene expression value of each group was normalized by control value, 14 weeks group. (**; *P* < 0.01, n = 5 mice/group, these were representative results from three independent experiments).

Twenty female C57BL/6 mice at the age of 6 weeks and twenty female 14 week-old mice were purchased from Samtako Co. (Gyeonggi-do, Korea). The skin of the left ear of each mouse was treated with *T*. *spiralis* ES-P (30 μg) or rTS 15-1c (30 μg) in PBS (total volume 50 μl) every day for 14 days, and that of the right ear was treated with PBS (Figs 1A and 7A). The mice were housed in a specific pathogen-free facility at the Institute for Laboratory Animals of Pusan National University.

### Cell culture and *in vitro* stimulation

In order to compare the expression level of type I collagen and their signal pathway related genes, mouse fibroblast (MEF) cells were used in this study because type I collagen was preferentially synthesized by two cell types, the osteoblast and the fibroblast [7]. MEF cells were isolated from C57BL/6 mouse fetuses 10 days after fertilization [8]. MEF cells were incubated in DMEM (Difco) with 5% FBS and 5 × 10^5^ cells were plated in 24-well plates and incubated overnight at 37°C in 5% CO_2_. The cells were treated with ES-P, boiled- ES-P, F1, F2, F3, boiled F3, TS 15-1c, and TS 15-1n (final conc. 1 μg/ml); ES-P with PMSF (serine protease inhibitor, final conc. 1 mM; Sigma-Aldrich, USA), F3 with PMSF for 2 hrs.

### Zymography

Gelatin-gel containing 0.2% gelatin was prepared from gelatin-stock solution. The proteins, *T*. *spiralis* ES proteins, TS 15-1c, and TS 15-1n were mixed with 2 × sample buffer (1 M Tris pH 6.8, 1% bromphenol blue, glycerol, β-mercaptoethanol), and the gel loaded with these proteins was run with 1 × Tris-Glycine SDS running buffer on 125 V for 2 hrs at 4°C. After running, the gel was washed to remove the SDS and re-natured proteinase activity with zymogram renaturing buffer (2.5% Triton X-100). The gel was developed with zymogram developing buffer (0.5 M Tris-HCl pH 7.6, 0.02 M NaCl, 0.5 mM CaCl_2_) for 30 min at room temperature. The gel was incubated with developing buffer at 37°C for 8 hrs. The gel was stained with Coomassie Blue R-250 for 30 min and distained with an appropriate destaining solution (Bio-Rad laboratories, Inc., USA).

### Real-time PCR

Homogenized ear tissues were mixed with TRIzol (Invitrogen, Germany), and RNA extraction and cDNA synthesis (Invitrogen, Germany) was performed in accordance with the manufacturer’s protocols. Expression levels of several genes were determined with real-time RT-PCR using the iCycler (Bio-Rad laboratories Inc., USA) real-time PCR machine. Primer sequences for *collagen I*, *TGF-β*, *smad2*, *smad3*, and *GAPDH*, and PCR conditions were identical to those mentioned in the previous study [5]. To evaluate variation of *Ts-15-1* gene expression during *T*. *spiralis* life cycle, total RNAs were extracted from new born larva, adult worm, muscle larva and *T*. *spiralis* infected mouse muscle (1, 2, and 4 weeks after infection) using TRIzol (Invitrogen, Germany), and cDNA synthesis (Invitrogen, Germany) was performed in accordance with the manufacturer’s protocols. Expression levels of several genes were determined with real-time RT-PCR using the iCycler (Bio-Rad laboratories Inc., USA) real-time PCR machine. The primer sequences for the putative trypsin (TS 15-1c), and *T*. *spiralis* GAPDH were 5′- TTG GAA TGA CGC TGA TTG -3′, 5′- GTG GCT TAT GAT GGT AGG AGA AT -3′ and 5′- CAG GTG CTG ATT ACG CTG TT -3′, R—5′- ACG CCA ATG CTT ACC AGA T -3′ respectively. Amplification of two genes was performed under the following conditions: 1 min 30 sec host start at 95°C, followed by denaturation at 95°C for 25 sec, primer annealing at 50 ~ 55°C for 20 sec, and elongation at 72°C and 30 sec for 40 cycles. Fluorescent DNA-binding dye SYBR was monitored after each cycle at 50 ~ 55°C. An iCycler multi-color real-time PCR detection system (Bio-Rad Laboratories) was used for estimation of expression levels. Then, using the Gene-x program (Bio-Rad Laboratories), relative expression of the gene was calculated as the ratio to a *T*. *spiralis* GAPDH gene.

### Immunoscreening of cDNA library

A cDNA library generated from 60,000 plaques forming units of *T*. *spiralis* muscle larvae was screened with the α-TS F3 antibody. Immunoscreening was performed using the SMART cDNA Library Construction Kit (Clontech, USA) in accordance with the manufacturer’s protocols. Briefly, after primary and secondary screening, positive plaques were picked and the phagemids were prepared by *in vivo* excision. The phagemids were transformed into XL1-Blue MRF cells. Clones were selected based on blue-white color selection of the colonies grown on LB-ampicillin agar plates. The plasmid harboring the cDNA inserts were then extracted using a plasmid DNA purification system (Cosmogenetech, Seoul, Korea). The cDNA inserts were then sequenced using the primer for T3 promotor (Cosmogenetech, DNA sequencing service, Seoul, Korea) and compared against the GenBank database.

### Construction of recombinant TS-15-1, TS 15-1c domain and TS 15-1n domain

Following confirmation of the PCR product sequences, *TS 15–1*, The *TS 15-1c* (C-terminal serine protease domain) and *TS 15-1n* (N-terminal serine protease domain), the genes were ligated with pET-28a vector (Novagen, USA). After gene ligation, the constructed plasmids were expressed in *Escherichia coli* BL21 (DE3, Novagen, USA). Pre-cultured cells were inoculated into Luria-Bertani broth containing kanamycin, and the cells were grown at 37°C until an OD_600_ of 0.5–0.6 was reached. Recombinant TS 15-1N and TS 15-1C expressions were induced addition of 0.5 mM isopropyl β-D-1-thiogalactopyranoside (IPTG) at 25°C for 16 h. The cells were harvested by centrifugation and the cell pellets were resuspended in buffer. A consisting of 50 mM Tris–HCl pH 7.5 and 200 mM NaCl. Cell disruptions were lysed by sonication on ice and the crude extracts were centrifuged to remove the cell debris. Ts15-1 N and C pellets were then sonicated in buffer including 50 mM Tris–HCl pH 7.5, 200 mM NaCl, and 6 M Urea on ice. The clear supernatant of the lysate was subjected onto the Ni–NTA column which had been pre-equilibrated with buffer A. The column was subsequently washed with buffer A containing imidazole, after which the bound proteins were eluted by varying the imidazole concentration (20–400 mM). The eluted proteins were analyzed using 10% SDS-PAGE. However, we could not get recombinant protein of TS 15–1 because very poor expression level.

### Production of polyclonal antisera for F3 fraction or TS 15-1c

Female four-week-old Wistar rats were purchased from Samtako Co. (Gyeonggi-do, Korea). Rats were immunized subcutaneously with a 1:1 mixture of the 250 μg F3 fraction of TS 15-1c protein (in 0.5 ml PBS) and 0.5 ml Freund’s complete adjuvant (#F5881, Sigma-Aldrich, USA) at 0 week. At 2 weeks the rat was given additional infections of the 250 μg F3 fraction or TS 15-1c protein with Freund’s incomplete adjuvant (#F5506, Sigma-Aldrich, USA). One week after their final booster, rats were sacrificed and serum was obtained.

### Real-time PCR

Homogenized ear tissues were mixed with TRIzol (Invitrogen, Germany), and total RNA extraction and cDNA synthesis (Invitrogen, Germany) was performed in accordance with the manufacturer’s protocols. Expression levels of several genes were determined with real-time RT-PCR using the iCycler (Bio-Rad laboratories Inc., USA) real-time PCR machine. Primer sequences for *collagen I*, *TGF-β*, *smad2*, *smad3*, and *GAPDH*, and PCR conditions were identical to those mentioned in the previous study [5]. Each gene expression levels were normalized with GAPDH gene expression.

### Collection of serum

Mice were killed at 0, 1, 2, and 4 weeks after *T*. *spiralis* infection and serum was obtained. Sera were stored at -20°C until used.

### Western blotting

Ten μg each of ES-P and F3 fraction (Fig 3) or 10 μg ES-P and total extract from *T*. *spiralis*, TS 15-1c (Fig 5B) or 10 μg of purified TS 15-1c antibody (Fig 5C) or 15 μg of each ear tissue samples (Fig 7) were separated on 10% acrylamide SDS-PAGE gel at 100 V for 90 min. Sweden), The loaded proteins were transferred onto a nitrocellulose membrane (Amersham Biosciences, Little Chalfont, UK) and blocked with 5% skim milk in TBST at 4°C overnight. Then, the membrane was incubated with primary antibody (polyclonal α-F3, α-TS 15-1c (1:500); time-course sera (1:1,000), α-TGF-β1 (1:1000; abcam, Carlsbad, CA, USA),;p-Smad2/3 (1:1000; Thermofisher science, Waltham, MC, USA),;α-mouse type I collagen (1:1000; abcam), and actin (1:5000, abcam)) in 5% skim milk in TBST for 2 hrs at room temperature. The secondary antibody, α-mouse or α-rat IgG-HRP conjugate (Sigma, Seoul, Korea) was used at 1:5,000 dilution for 1 hr at room temperature. HRP was detected using an ECL substrate (Amersham Biosciences, Uppsala, Sweden), analyzed using the LAS 3000 machine. (Areas of the detected bands were determined and compared by Image J software).

### Immunofluorescence

Paraffin-embedded *T*. *spiralis* infected or non-infected mouse muscle tissue were de-paraffinized and hydrated. For antigen retrieval, slides were immersed in citrate buffer (0.01 M, pH 6.0) and heated twice in a microwave (700 W or ‘high’) for 5 min. Slides were then quenched with endogenous peroxidase by incubation in a 3% hydrogen peroxide solution for 5 min and were washed three times in PBS for 5 min each. Slides were immuno-stained with primary antibody (α-TS 15-1c antibody that was produced according to the polyclonal antisera method; 1:500 dilution) at 4°C overnight. After primary antibody incubation, slides were washed three times in PBS for 5 min each and were incubated with secondary antibody, the Alexa Fluor 594 goat anti-rat IgG secondary antibody (1:500; Invitrogen, USA) was applied for 1 h at 24°C. The slides were washed in PBS and mounted with Permount (Fisher Scientific, Pittsburgh, PA, USA). Confocal images of stained muscle tissue were examined under an inverted fluorescence microscope.

### Statistical analysis

All experiments were performed three times for confirmation of statistical significance. Mean ± standard deviation (SD) was calculated from data collected from individual mice. Significant differences were determined using one-way or two-way analysis of variance. Statistical analysis was performed with GraphPad Prism 5.0 software (GraphPad Software Inc., CA, USA).

### Ethics statement

The study was performed with approval from the Pusan National University Animal Care and Use Committee (IACUC protocol approval; PNU-2016-1175), in compliance with ‘‘The Act for the Care and Use of Laboratory Animals” of the Ministry of Food and Drug Safety, Korea. All animal procedures were conducted in a specific pathogen-free facility at the Institute for Laboratory Animals of Pusan National University.

## Results

### Type I collagen gene expression in an aging mouse model

In order to understand the collagen gene inducing effect of ES-P, transcription and protein expression levels of type I collagen and TGF-β1 signaling related proteins were compared in ear tissues of 6 and 14 week-old mice that had or had not received ES-P treatment (Fig 1A). The expression levels of the *collagen I* and *TGF-β1* genes of the 14 week-old mice were significantly decreased compared to those of the 6 weeks mice. However, those of the ES-P treated 14 week-old mouse group were significantly increased compared to un-treated 14 week-old mice (Fig 1B).

### Fractionation of ES-P and evaluation of collagen gene induction of major fractions

**Fig 2:**
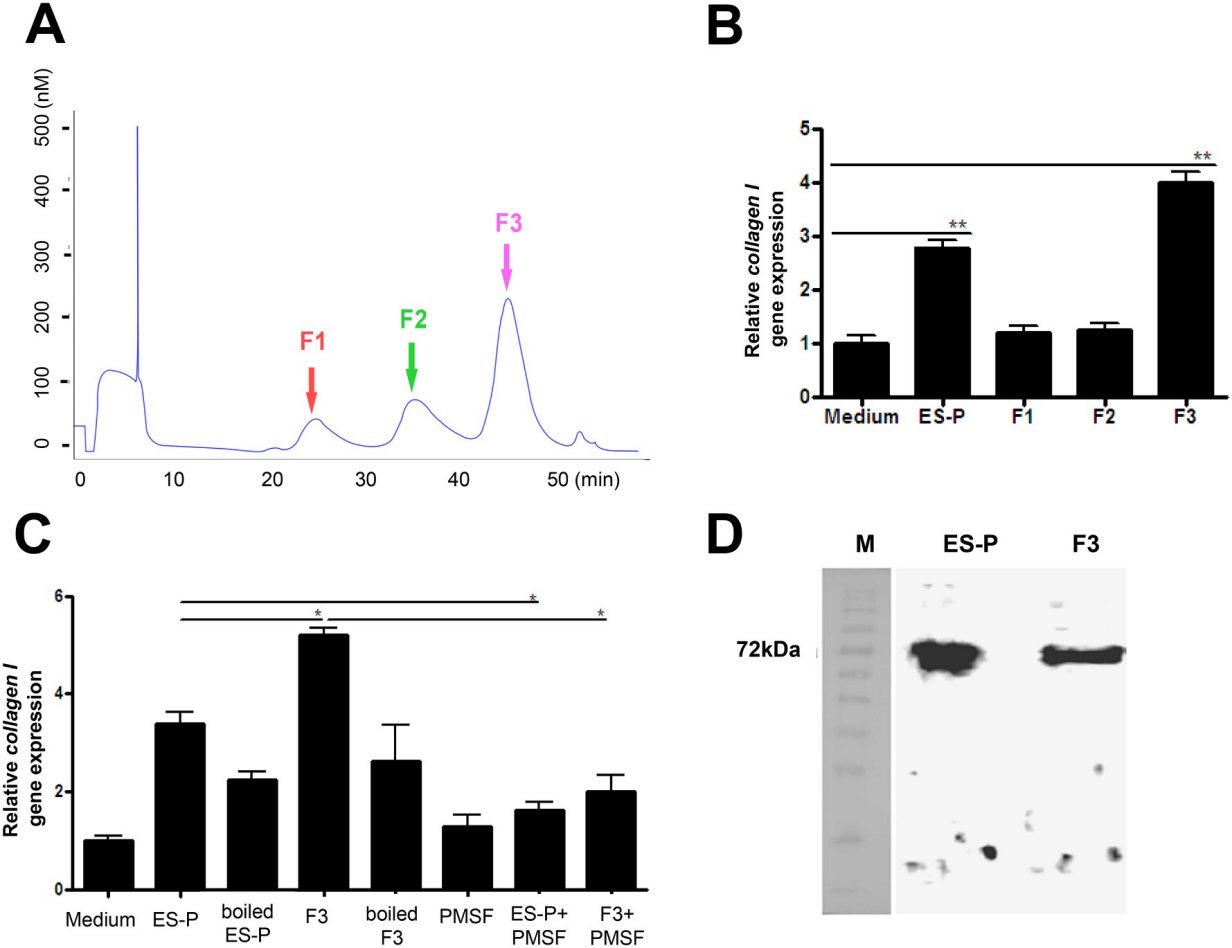
Fractionation of the ES-P by chromatography and type I collagen expression analysis. (A) Chromatograhic profile of ES-P conducted by gel filtration chromatography. (B) Type I collagen gene expression levels were compared after ES-P or fractionation treatment. The collagen gene expression levels of each group were normalized with medium group. (Medium; cell culture medium, ES-P; *T*. *spiralis* ES proteins, F1; F1 fraction, F2; F2 fraction, F3; F3 fraction). (C) To confirm the collagen inducing ability was related with protease activity. (Boiled ES-P; treatment with *T*. *spiralis* ES protein boiled, boiled-F3; treatment with F3 boiled for 10 min at 90°C, PMSF; only PMSF treatment, ES-P+PMSF; *T*. *spiralis* ES proteins and PMSF treatment, F3+PMSF; F3 fraction and PMSF treatment). (D) Western blot analysis of ES-P and F3 with polyclonal α-F3 antibody. (*; *P* < 0.05, **; *P* < 0.01, these were representative results from three independent experiments).

To identify type I collagen inducing factors from the *T*. *spiralis* ES-P, the ES-P was fractionated to several fractions including three big fractions by gel filtration chromatography. The three major fractions obtained were named F1 (about 100 kDa– 140 kDa), F2 (about 80 kDa—120 kDa), and F3 (about 50 kDa -90 kDa) respectively (Fig 2A). In order to determine which major fraction had collagen gene expression inducing factors, each fraction was used to treat MEF cells, and expression levels of the type I collagen gene were measured. After F1, F2, and F3 treatment, only F3 treated MEF cells had significantly increased type I collagen gene expression (Fig 2B). Moreover, expression levels of *collagen I* in F3 treated MEFs was higher those of ES-P treated MEFs (Fig 2B).

### Protein activity evaluation and size determine of F3

In order to determine whether the collagen inducing ability of F3 is related with serine protease activity, we evaluated the collagen inducing ability of F3 following pre-treatment with a serine protease inhibitor, PMSF, on MEF cells. The *collagen I* gene expression levels were significantly decreased in MEF cells pre-treated with PMSF, the serine protease inhibitor. In addition, the gene expression levels were also not increased when treated with boiled F3 (Fig 2C). In order to confirm the existence and expression levels of F3 in ES-P, α-F3 polyclonal antibody was used against ES-P and F3 in a western blot analysis. The presence of a strong band was observed at 60–80 kDa in both the ES-P and F3 samples (Fig 2D).

### Immunoscreening of a cDNA library from *T*. *spiralis* muscle larva by α-F3 antibody

In order to identify the collagen gene inducing factors from *T*. *spiralis* ES-P, immunoscreening was conducted against the *T*. *spiralis* muscle larvae Express cDNA library with the α-F3 antibody. Thirty-five positive plaques were detected in primary screening, among them, 10 plaques were confirmed by second screening (S1 Fig). These plaques were amplified and processed in an *in vivo* excision step. All the insert DNA from the 10 positive clones were sequenced and their amino acid sequences were determined. Two insert DNA fragments (TS 15–1 and TS 15–2) were similar to a putative trypsin of *T*. *spiralis* with 90% identity. Another clone (TS 15–3) was matched to a nuclear receptor-binding protein of *T*. *spiralis* with 35% of identity. Another clone (TS-16-1) was matched to a putative BTB/POZ domain protein of *T*. *spiralis* with 63% identity. The remaining 6 insert clones were not matched with any previously known genes.

### Molecular characterization of *TS 15–1*

**Fig 3:**
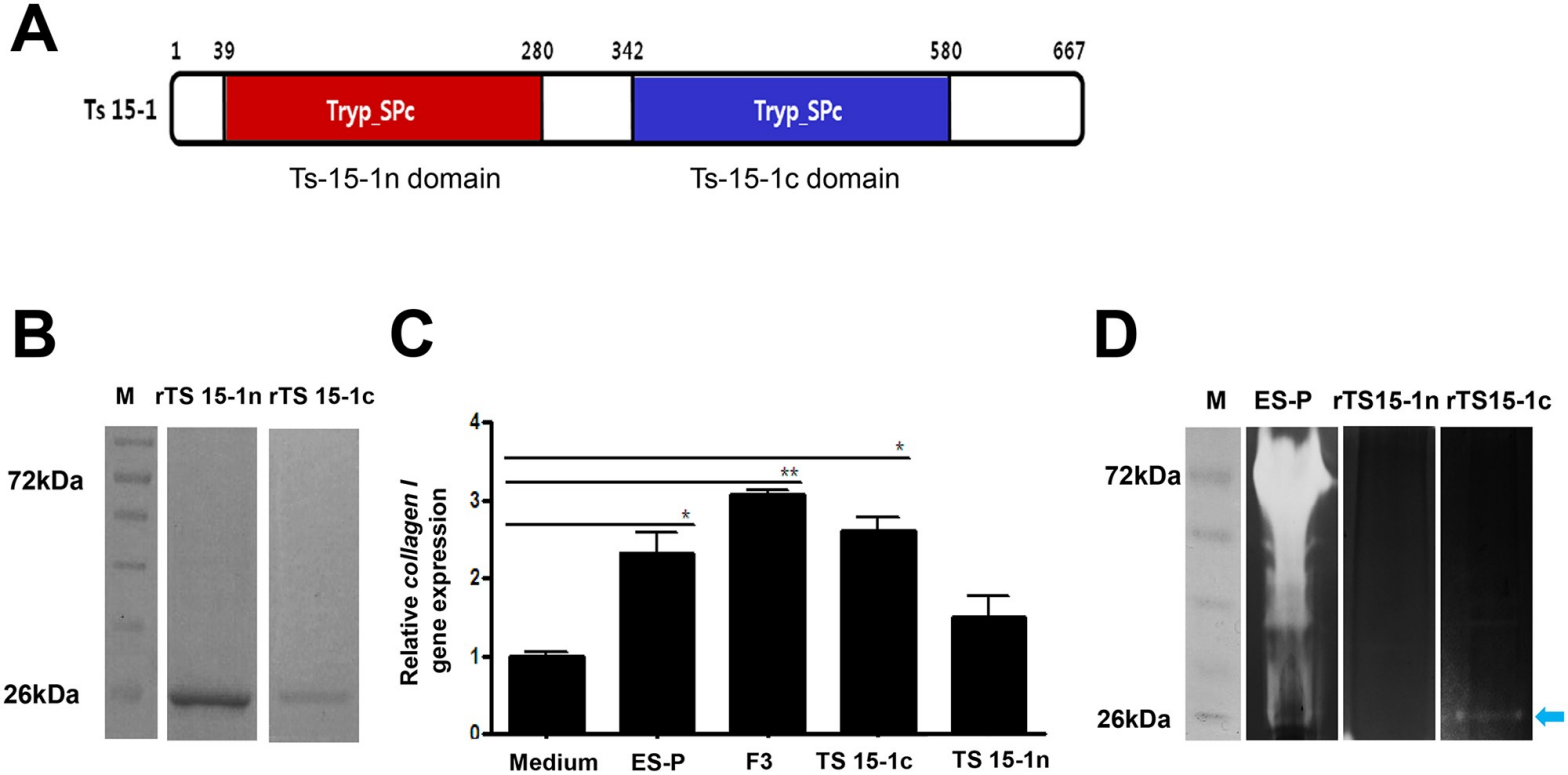
Molecular structure and characterization of TS 15–1. (A) Schematic diagram showing the domains of the full-length TS 15–1. TS 15–1 consists of two trypsin domains. (red; N-terminal Tryp_SPc domain, blue; C-terminal Tryp_SPc domain). (B) SDS-PAGE loading of recombinant proteins rTS 15-1n and rTS 15-1c. (C) Comparison of collagen gene inducing abilities of the recombinant proteins and ES-P, the expression level of test samples was compared with control group. The expression levels of *collagen I* were analyzed by real-time PCR. The gene expression value of each group was normalized by medium group’s value. (D) Proteolytic activity evaluation of two recombinant proteins. The ES-P, rTS 15-1n, and rTS 15-1c was used for gelatin-zymography to determine protease activity. The white region was indicated that digested gelatin in the gel by protease activity of the samples. Arrow indicates digested gelatin by rTS-15-1c protease activity (*; *P* < 0.05, **; *P* < 0.01, these were representative results from three independent experiments).

Collagen inducing factors in ES-P and F3 had serine protease activity and were measured to be about 60–72 kDa in size. After evaluation of the size and serine protease activity of positive clone matched genes, the *TS 15–1* gene was selected for downstream identification of the collagen inducing factor. The *TS 15–1* fragment was 2,004 bp long and encoded a 667 amino acid protein, and the molecular weight and pI was calculated as 71.6 kDa and 8.83. The deduced TS 15–1 protein has two conserved catalytic motifs, an N-terminal Tryp_SPc domain (TS 15-1n) and a C-terminal Tryp_SPc domain (TS 15-1c) (Fig 3A). The TS 15-1n and TS 15-1c peptides were composed of 238 amino acids and 239 amino acids respectively, and the molecular weight was calculated to be 26.1 kDa and 26.2 kDa respectively. Unfortunately, collection of recombinant full length of TS-15 protein was very difficult because its expression level was very low. We conducted recombinant protein expression of the N and C terminal domains (about 26 kDa, Fig 3B) and evaluated their collagen gene inducing ability. *collagen I* expression levels in the recombinant TS 15-1c protein treated cells were significantly increased compared to a media control. However, recombinant TS 15-1n protein treated cells were not significantly changed compared to those of medium treated cells (Fig 3C). In order to know the protease activity of both recombinant proteins, zymogram analysis was conducted. A collagen digested clear zone was detected around ~26 kDa in the recombinant TS 15-1c protein lane, but the clear zone was not detected with the recombinant TS 15-1n protein (Fig 3D).

### Molecular structure of TS 15-1c & TS 15-1n

**Fig 4:**
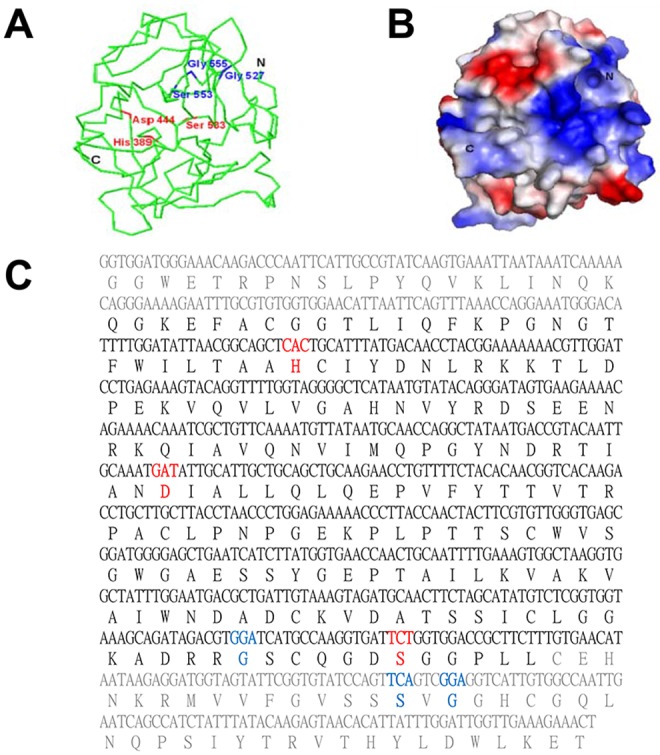
Representation of the 3D structure of TS 15-1c and prediction of active sites. (A) The modeled structure of TS 15-1c is shown as a Cα trace representation, and (B) a surface representation. The relative distribution of the surface charge is shown with acidic regions in red, basic regions in blue and neutral regions in white. (C) Amino acid sequence of the C-terminal domain of TS 15–1. Predicted active sites (H389, D444, and S533) and substrate binding sites (G527, S553, and G555) are shown in blue and red, respectively.

In this study, we determined a molecular model by homology modeling based on the structure of another serine protease (PDB ID: 1KYN, 1–235). In TS 15-1c (G342—T580), predictions of the active sites (H389, D444, and S533) and the substrate binding sites (G527, S553, and G555) are shown in blue and red letters, respectively (Fig 4A). Interestingly, these results indicated that these sites interact with inhibitors and ligands. Most residues in these regions had negative charges in a globular fold (Fig 4B). Surprisingly, both TS 15-1n and granzyme B structures were shown to have very similar folding patterns (S2 Fig). The TS 15-1n is approximately 35% homologous to granzyme B (21–247). We found TS-15-1c and TS 15-1n molecular 3D model was quite different each other.

### Expression and localization of TS-15 in the *T*. *spiralis* life cycle

**Fig 5:**
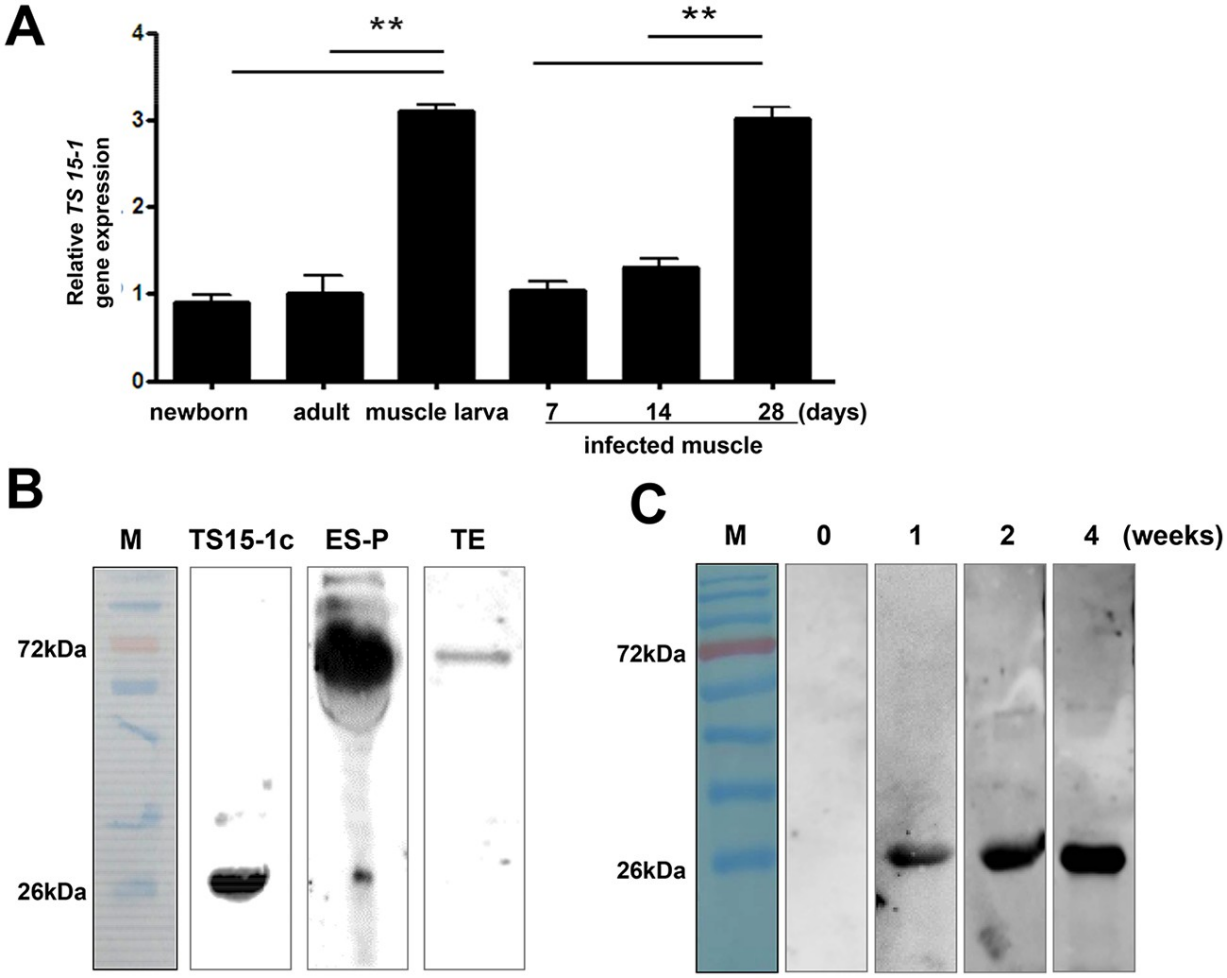
Expression levels of *TS15-1* during developmental stages and *T*. *spiralis* infected muscle, and evaluation of TS15-1 antigenicity. (A) Larva from each stage, the adult worm, and *T*. *spiralis* infected muscle (7, 14 and 28 days after infection) were collected and *TS 15–1* gene expression levels were analyzed with real-time PCR analysis. (**; P< 0.01, these were representative results from three independent experiments). (B) The polyclonal α-TS 15-1c antibody was detected on TS15-1c, ES-P, and TE from *T*. *spiralis* or (C) sera of *T*. *spiralis* infected mice at 1, 2 and 4 weeks by western blot analysis. (M, molecular marker).

**Fig 6:**
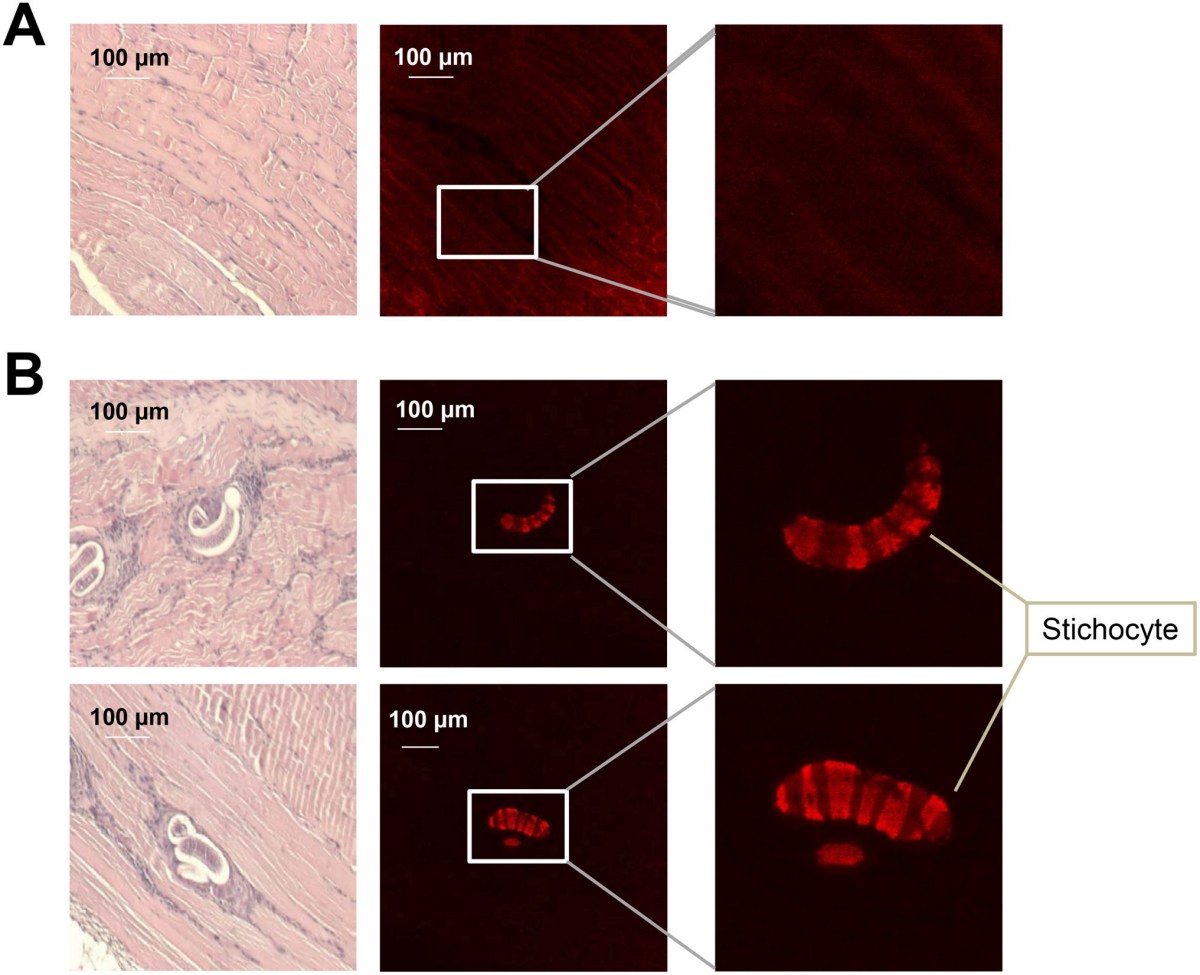
Localization of TS 15–1 in muscle stage larvae. Serial sections of muscle larvae were detected with α-TS 15-1c antibody. The TS 15–1 protein was localized in stichosome (arrow); (A; non-infected muscle section, B; infected muscle section).

In order to determine when the *Ts 15–1* mRNA was the most highly expressed in the *T*. *spiralis* life cycle, real-time PCR was performed on new born larvae, adult worms, muscle stage larvae of *T*. *spiralis*, and during the *T*. *spiralis* infection period (at 0, 7, 14, 28 days after infection). As the results show, the *Ts 15–1* gene was the most highly expressed in muscle stage larvae, and its expression is also highly elevated 28 days after infection (Fig 5A). In order to know whether TS 15–1 was secreted from parasites, an α-TS 15-1c antibody was produced and was reacted with *T*. *spiralis* ES-P and total extract. The TS 15-1c antibody strongly reacted with proteins around 72 kDa in ES-P and slightly reacted with a total extract at the same size (Fig 5B). Furthermore, to know whether TS 15-1c has antigenicity or not, *T*. *spiralis* infected mice sera (0, 1, 2, and 4 weeks after infection) were reacted with recombinant TS 15-1c protein (Fig 5C). rTS 15-1c most strongly reacted with mouse serum collected 4 weeks after infection. To know where TS 15–1 is secreted in the parasite, α-TS 15-1c antibody was reacted against serial sections of the *T*. *spiralis* infected muscle using immunohistochemical methods. α-TS 15-1c antibody strongly reacted with only the ladder shapes structure around the esophagus in muscle stage larvae that appear to be stichocytes (Fig 6).

### Evaluation of the TS 15-1c protein type I collagen inducing ability

**Fig 7:**
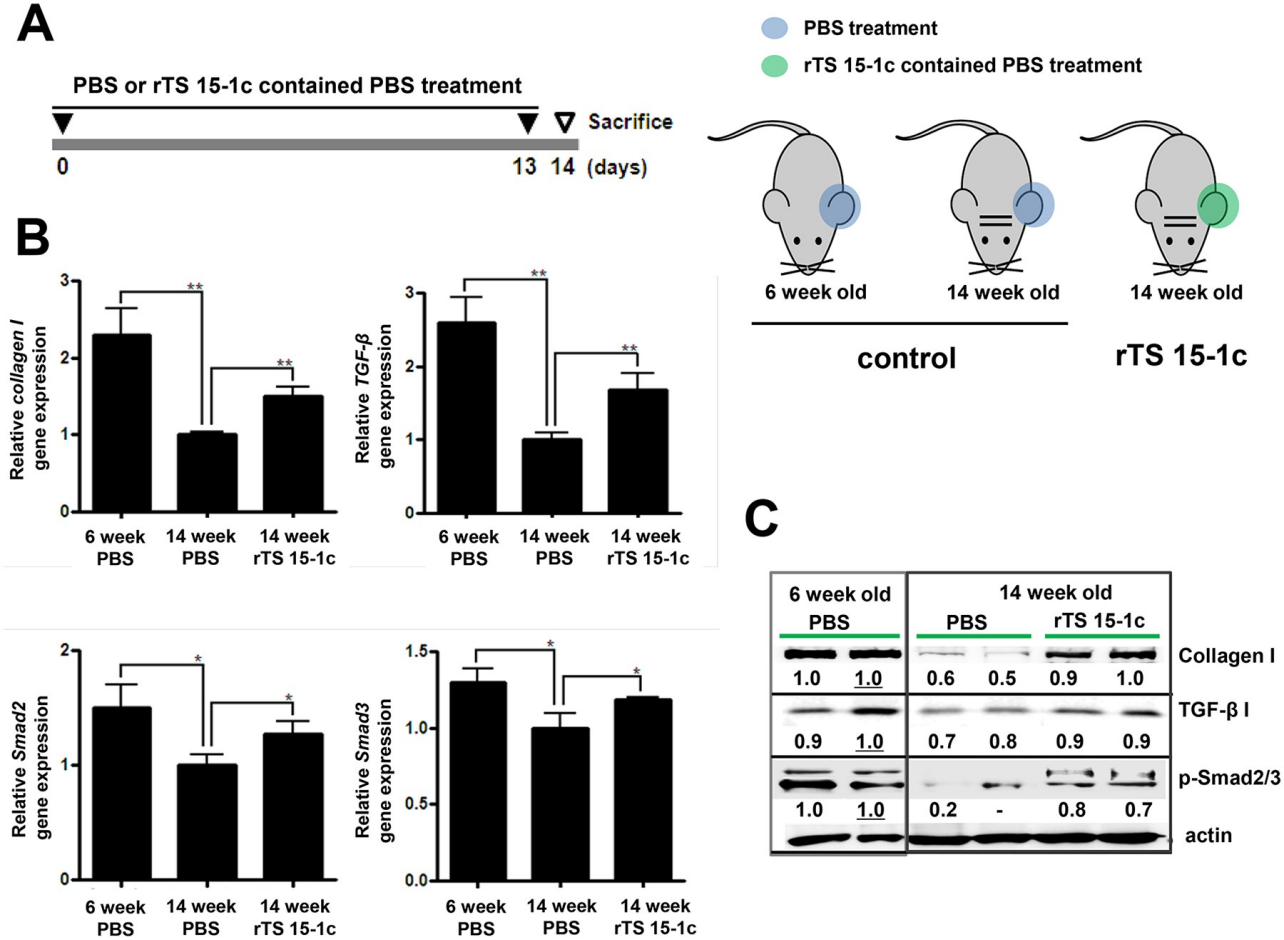
Evaluation of collagen I and TGF-β signaling pathway-related protein expression after treatment of recombinant TS 15-1c protein on natural aged mice. (A) Schematic schedule of the purified TS 15-1c treatment. The PBS (blue color) and 30 μg rTS 15-1c contained PBS (green color) were treated daily on ears of 6 week or 14 week old mice. The total RNA was extracted from the sample treated ears, and cDNAs were synthesis according to manufactural protocols. (B) The gene expression levels of *collagen I*, *TGF-β1*, and *Smad2/3* were analyzed via real-time PCR. (*; *P* < 0.05, **; *P* < 0.01, n = 3 to 5 mice/group, these were representative results from three independent experiments). (C) The gene expression levels of *collagen I*, *TGF-β1*, and *Smad2/3* were analyzed by western blot analysis.

In order to know whether TS 15-1c had type I collagen elevating ability, we applied TS 15-1c to the ear skin of 6 week- and 14 week-old mice and evaluated the expression levels of *collagen I*, *Smad2/3*, and *TGF-β1* (Fig 7A). All of the tested genes’ expression levels, including the *collagen I* gene of the 14 week-old mice, were significantly lower than those in the 6 week-old mice. However, after 14 TS 15-1c treatments on 14 week-old mice, *collagen I*, *Smad2/3*, and *TGF-β1* gene expression levels in these mice were significantly increased compared with non-treated mice of the same age (Fig 7B). We investigated protein levels of type I collagen, the phosphorylation form of Smad2/3, and levels of TGF-β1 in TS 15-1c treated 14 week-old mice and the protein levels were considerably recovered relative to those of the non-treated 14 week-old mice (Fig 7C).

## Discussion

In this study, we identified the host collagen inducing factor from *T*. *spiralis*, named it TS 15–1, and confirmed its serine protease activity and ability to elevate type I collagen, TGF-βI, and related signal proteins (Smad2/3) on a transcriptional and protein level. In addition, we found that it was secreted outside the parasite and elicited specific antibody production from the host immune system. In a previous study, we revealed that the ES-P of *T*. *spiralis* could induce collagen production of host muscle tissue during the infection period, and that it was closely related with serine protease activity [5].

We can carefully suggest that TS 15–1 is one of the key collagen inducing factors in ES-P revealed in our previous study. Although we could not demonstrate that TS 15–1 is one of the key molecules in the collagen capsules around the nurse cell formation step, it might be one of the central factors for collagen capsule formation. This is because *TS 15–1* gene expression level was the highest during the *T*. *spiralis* muscle larva stage and its specific antibodies could be detected in mouse serum from 1 week up to 4 weeks after a *T*. *spiralis* infection (Fig 5). During the nurse cell formation period (1 week–4 weeks), *T*. *spiralis* might strongly secrete TS 15–1 to induce collagen capsule synthesis by the host muscle cell.

Parasite secretory proteases might have important functions in modulating the interactions between parasites and hosts because of their particular roles in the invasion of host tissues, parasite nutrition, and evasion of host immune responses [9–12]. A trypsin-like serine protease of parasites could be involved in host immune regulation [13–15]. Serine proteases in nematodes are known to be involved in invasion into host cells and tissues and are likely to be important in molting. TS 15–1 was revealed to be a serine protease, trypsin like protein, because its activity was inhibited by PMSF (Fig 2C) and it was composed two domains which were very similar but not identical to each other (Fig 3A). Several secreted serine proteases have been identified among *T*. *spiralis* ES proteins, including the 69 kDa putative serine protease TsSerP (two trypsin-like domains), the 45 kDa serine protease TspSP-1, and a 35.5 kDa serine protease [9, 11, 16–18]. Most of these have strong antigenicity, specific antibodies for them are easily detected experimentally in infected animal sera, and they have one or two trypsin like domains [9, 11, 16]. Most secreted proteases could elevate their specific antibodies during nematodiasis [16, 19]. Trap et al., reported the identification of the putative serine protease, TsSerP, isolated from the *T*. *spiralis* adult-newborn larvae stage. It has two trypsin-like serine protease domains flanking a hydrophilic domain, which is the same structure as TS 15–1. Immunohistochemistry analysis revealed that TsSerP was located on the inner layer of the cuticle and esophagus of the parasite, TS 15–1 was also detected on the inner layer of the cuticle and stichocytes in this study (Fig 6). These two serine proteases of *T*. *spiralis* might have similar functions, although the function of TsSerP was not clearly revealed [9].

In this study, it was revealed that TS 15–1 could elevate collagen expression via the TGF-β1 signaling pathway in host tissue of normal aged mice. Because, the recombinant TS 15-1c protein could elevate collagen I production and the TGF-βI signaling pathway related to Smad2/3 proteins (Fig 7). Type I collagen expression is closely related with the TGF-βI/Smad2/Smad3 signaling pathway [20–22]. This characteristic could be used for therapeutic effects including wound healing and cosmetic usefulness with wrinkle reduction. The various serine proteases may participate in physiological or pathological processes, like tissue repair, vascular remodeling, and wound healing, which depend on cell proliferation and migration [23, 24]. Type I collagen is the major structural protein in the skin. Collagen destruction is thought to underlie the appearance of aged skin and changes resulting from chronic sun exposure [25]. Ultraviolet irradiation from the sun has deleterious effects on human skin including cancer, photo-aging, and intrinsic aging [26]. TGF-β/Smad pathway is the major regulator of collagen homeostasis and plays a crucial role in dermal fibrosis [27, 28]. TGF-β is the most potent direct stimulator of collagen production. Moreover, TGF-β is central to the process of wound healing and fibrosis formation in skin [29, 30]. It is well understood that activation of TGF-β signaling pathways stimulus the Smad family downstream via phosphorylation. Wound healing is a well-orchestrated process, where numerous factors are activated or inhibited in a sequence of steps [31]. Numerous signaling pathways are involved, among of them, the TGF-β1/Smad pathway is representative and well known to participate in the wound healing process [31]. Hozzein et al., suggested that topical application of propolis would promote the wound healing process by promoting TGF-β/Smad signaling, leading to increased expression of collagen type I [32]. The gradual loss of collagen in skin with aging results in wrinkles and other signs of skin aging [33]. The content of type I collagen, the major collagen in the skin and a marker of collagen synthesis, is deceased by 68% in old skin versus young skin, and cultured young fibroblasts synthesize more type I collagen than old cells [33]. In addition, a possible influence of collagen membrane on extracellular matrix synthesis was addressed using analysis of TGF-β1 and Smad2/3 complex [34, 35].

In conclusion, we identified a host collagen inducing factor from ES-P using immune screening methods and demonstrated the molecular/genetic characteristics and function of TS 15-1c. Further study will be required to understand the detailed mechanisms for receptors in the host cells, and to identify the minimal structure that can induce collagen for cosmetic and medical purposes.

## Supporting information

S1 Fig Result of immunoscreening of *T*. *spiralis* cDNA library by α-F3 antibody. After *T*. *spiralis* cDNA containing phages were mixed with *E*. *coli*, the plaques were incubated with NC membranes for 4 hrs. The membranes were reacted with α-F3 antibody (1:500) as the primary antibody and α-rat IgG antibody conjugated with HRP was reacted as secondary antibody. After adding of 3,3'-diaminobenzidine (DAB), colored spots were compared with original plates. Positive plaques were amplified and re-analyzed by the same method.
(PPTX)

S2 Fig Representation of the 3D structure of TS 15-1n and prediction of active sites. The modeled structure of TS 15-1n is shown as a surface representation (A), and a Cα trace representation (B). In TS-15-1n, predictions of the active sites (G227, S252, and H254) and the substrate binding sites (R88, D142, and S233) are shown in blue and red letters, respectively. The relative distribution of the surface charge is shown with acidic regions in red, basic regions in blue and neutral regions in white. Amino acid sequence of the N-terminal domain of TS 15–1 (C).
(PPTX)
