# Supplementary material for: Identification of a host collagen inducing factor from the excretory secretory proteins of Trichinella spiralis
Source: PLoS Negl Trop Dis. 2018 Nov 1;12(11):e0006516. doi: 10.1371/journal.pntd.0006516 (PMC6233931; doi:10.1371/journal.pntd.0006516)
Supplement: S1 Fig — After T. spiralis cDNA containing phages were mixed with E. coli, the plaques were incubated with NC membranes for 4 hrs. The membranes were reacted with α-F3 antibody (1:500) as the primary antibody and α-rat IgG antibody conjugated with HRP was reacted as secondary antibody. After adding of 3,3'-diaminobenzidine (DAB), colored spots were compared with original plates. Positive plaques were amplified and re-analyzed by the same method. (PPTX) [file pntd.0006516.s001.pptx]

## Slide 1
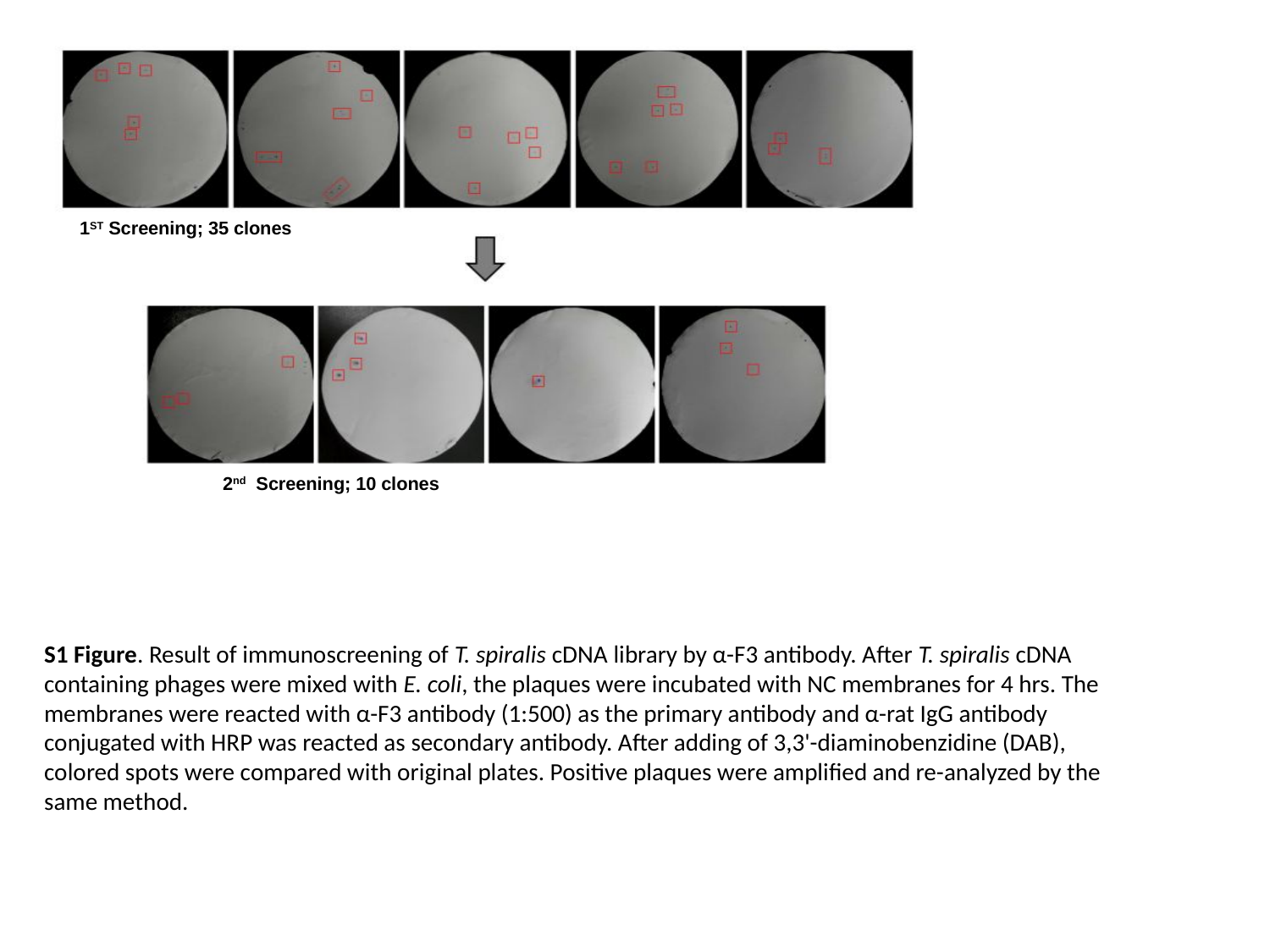

1ST Screening; 35 clones
2nd Screening; 10 clones
S1 Figure. Result of immunoscreening of T. spiralis cDNA library by α-F3 antibody. After T. spiralis cDNA containing phages were mixed with E. coli, the plaques were incubated with NC membranes for 4 hrs. The membranes were reacted with α-F3 antibody (1:500) as the primary antibody and α-rat IgG antibody conjugated with HRP was reacted as secondary antibody. After adding of 3,3'-diaminobenzidine (DAB), colored spots were compared with original plates. Positive plaques were amplified and re-analyzed by the same method.
