# Supplementary material for: Identification of a host collagen inducing factor from the excretory secretory proteins of Trichinella spiralis
Source: PLoS Negl Trop Dis. 2018 Nov 1;12(11):e0006516. doi: 10.1371/journal.pntd.0006516 (PMC6233931; doi:10.1371/journal.pntd.0006516)
Supplement: S2 Fig — The modeled structure of TS 15-1n is shown as a surface representation (A), and a Cα trace representation (B). In TS-15-1n, predictions of the active sites (G227, S252, and H254) and the substrate binding sites (R88, D142, and S233) are shown in blue and red letters, respectively. The relative distribution of the surface charge is shown with acidic regions in red, basic regions in blue and neutral regions in white. Amino acid sequence of the N-terminal domain of TS 15–1 (C). (PPTX) [file pntd.0006516.s002.pptx]

## Slide 1
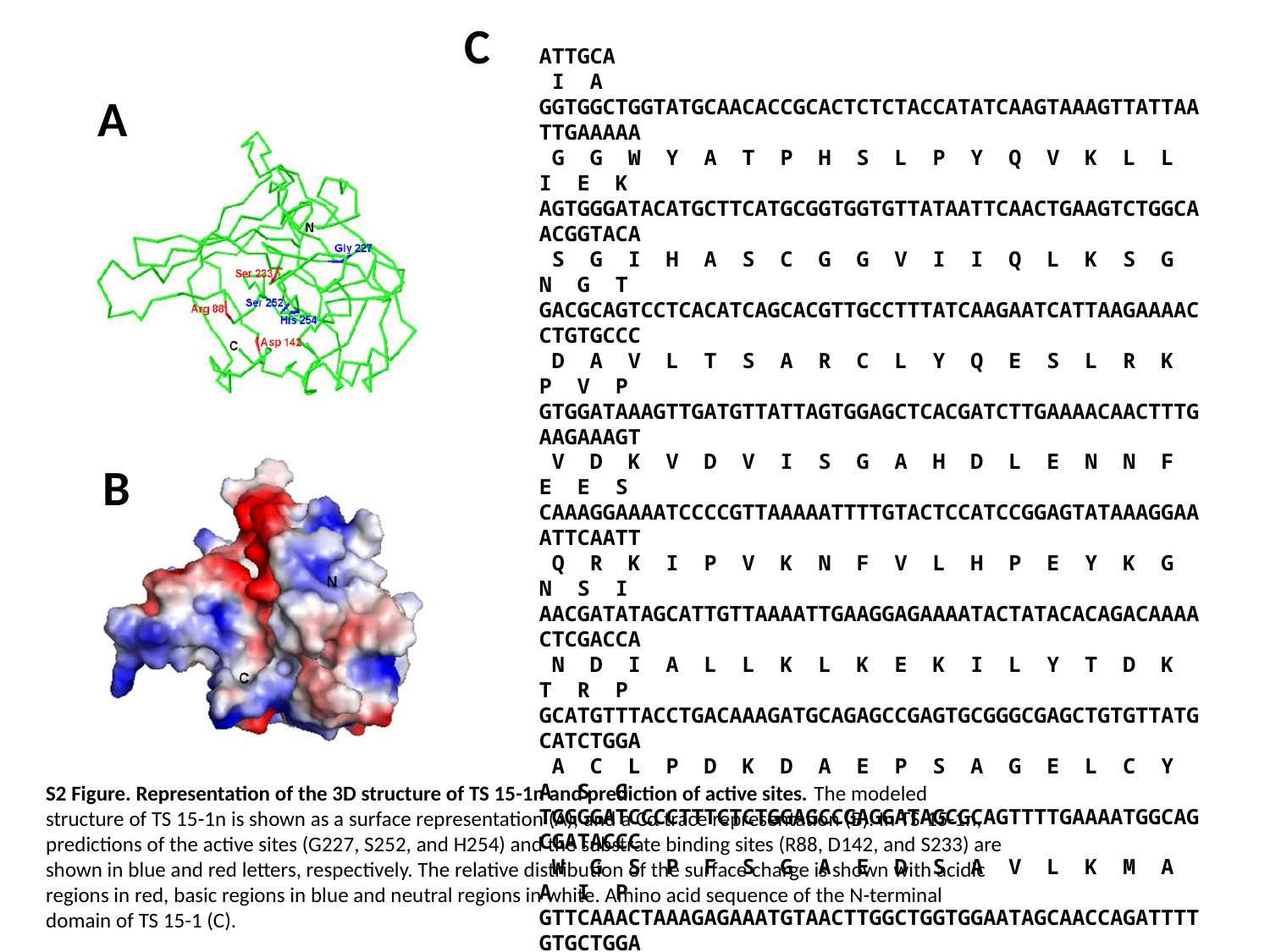

C
ATTGCA
 I A
GGTGGCTGGTATGCAACACCGCACTCTCTACCATATCAAGTAAAGTTATTAATTGAAAAA
 G G W Y A T P H S L P Y Q V K L L I E K
AGTGGGATACATGCTTCATGCGGTGGTGTTATAATTCAACTGAAGTCTGGCAACGGTACA
 S G I H A S C G G V I I Q L K S G N G T
GACGCAGTCCTCACATCAGCACGTTGCCTTTATCAAGAATCATTAAGAAAACCTGTGCCC
 D A V L T S A R C L Y Q E S L R K P V P
GTGGATAAAGTTGATGTTATTAGTGGAGCTCACGATCTTGAAAACAACTTTGAAGAAAGT
 V D K V D V I S G A H D L E N N F E E S
CAAAGGAAAATCCCCGTTAAAAATTTTGTACTCCATCCGGAGTATAAAGGAAATTCAATT
 Q R K I P V K N F V L H P E Y K G N S I
AACGATATAGCATTGTTAAAATTGAAGGAGAAAATACTATACACAGACAAAACTCGACCA
 N D I A L L K L K E K I L Y T D K T R P
GCATGTTTACCTGACAAAGATGCAGAGCCGAGTGCGGGCGAGCTGTGTTATGCATCTGGA
 A C L P D K D A E P S A G E L C Y A S G
TGGGGATCCCCTTTCTCTGGAGCCGAGGATAGCGCAGTTTTGAAAATGGCAGCGATACCC
 W G S P F S G A E D S A V L K M A A I P
GTTCAAACTAAAGAGAAATGTAACTTGGCTGGTGGAATAGCAACCAGATTTTGTGCTGGA
 V Q T K E K C N L A G G I A T R F C A G
GGAAGCTTTGGAGGACACGGAATTTGTGACGGTGATTCAGGAGGACCATTAACATGCGAA
 G S F G G H G I C D G D S G G P L T C E
AGAAATGGAAAATTGGTTGTATTTGGCATATCAAGTGGACATACAGGCCTCTGTGGTCAA
 R N G K L V V F G I S S G H T G L C G Q
TATGGCAAACCAGGAATTTTTACAAAAGTATCATCCTTTTTGGATTGGATTAAAAAAACT
 Y G K P G I F T K V S S F L D W I K K T
GATACA
 D T
A
B
S2 Figure. Representation of the 3D structure of TS 15-1n and prediction of active sites. The modeled structure of TS 15-1n is shown as a surface representation (A), and a Cα trace representation (B). In TS-15-1n, predictions of the active sites (G227, S252, and H254) and the substrate binding sites (R88, D142, and S233) are shown in blue and red letters, respectively. The relative distribution of the surface charge is shown with acidic regions in red, basic regions in blue and neutral regions in white. Amino acid sequence of the N-terminal domain of TS 15-1 (C).
